# Comparison of methodologies to define hemodialysis patients hyporesponsive to epoetin and impact on counts and characteristics

**DOI:** 10.1186/1471-2369-14-44

**Published:** 2013-02-20

**Authors:** David T Gilbertson, Yi Peng, Thomas J Arneson, Stephan Dunning, Allan J Collins

**Affiliations:** 1 Chronic Disease Research Group, Minneapolis Medical Research Foundation, 914 South 8th Street, Suite S2.213, MN 55404, Minnesota, USA; 2 Department of Medicine, University of Minnesota, Minneapolis, MN, USA

**Keywords:** Epidemiology, Epoetin, Hemodialysis

## Abstract

**Background:**

Some hemodialysis patients require large doses of erythropoiesis-stimulating agents (ESAs) to manage anemia. These patients, termed “ESA hyporesponsive,” have been characterized using various definitions. We applied three definitions of hyporesponsiveness to a large, national cohort of hemodialysis patients to assess the impact of definition on counts and on characteristics associated with hyporesponsiveness.

**Methods:**

We studied point-prevalent hemodialysis patients on May 1, 2008, with Medicare as primary payer, who survived through December 31, 2008. Included patients received recombinant human erythropoietin (EPO) in each month, August-December. Hyporesponsiveness definitions were: above the ninetieth percentile of total monthly EPO dose; above the ninetieth percentile of total monthly EPO dose divided by weight in kg; above the ninetieth percentile of total monthly EPO dose divided by hemoglobin level. Hyporesponsiveness was further classified as chronic, acute, or other. Comorbid conditions were assessed before and concurrent with the hyporesponsive period.

**Results:**

Women, African Americans, and patients aged <40 years, with cause of renal failure other than diabetes or hypertension, or longer dialysis duration, were more likely to be hyporesponsive. Antecedent comorbid conditions most predictive of any subsequent hyporesponsiveness were congestive heart failure, peripheral vascular disease, other cardiac disease, gastrointestinal bleeding, and cancer. Concurrent comorbid conditions most strongly associated with any hyporesponsiveness were gastrointestinal bleeding and cancer. All conditions were somewhat more likely when ascertained concurrently. Comorbidity burdens were lowest for non-hyporesponsive patients.

**Conclusions:**

As associations were similar between patient characteristics and three methods of characterizing EPO hyporesponsiveness, the simplest definition using EPO dose can be used.

## Background

Endogenous erythropoietin production is greatly reduced for patients receiving maintenance hemodialysis, who generally require regular administration of exogenous erythropoietin or transfusions. A subset of these patients receive very large doses of erythropoiesis-stimulating agents (ESAs) as part of a strategy for anemia management. The term “ESA hyporesponsive” has been used to refer to patients who need high doses of ESAs to increase and maintain their hemoglobin levels. This phenomenon is generally believed to be due to a variety of factors, including absolute or functional iron deficiency and inflammation. Patients can experience short-term acute episodes of hyporesponsiveness, or longer more chronic episodes. There does not appear to be a standardized definition of ESA hyporesponsiveness. However, a number of studies have investigated various aspects of hyporesponsiveness, using a variety of definitions and producing different estimates of proportions of hyporesponsive patients and factors associated with hyporesponsiveness. Management of anemia is expensive for these patients, and interest in increasing treatment effectiveness and efficiency is growing, particularly with the January 2011 implementation of the new Medicare dialysis reimbursement bundle, which includes ESAs. In this study, we sought to investigate different definitions of ESA hyporesponsiveness and their associations with patient characteristics. We applied three different definitions of hyporesponsiveness to the same large, national cohort of hemodialysis patients to assess the impact of definition on counts and on patient characteristics associated with hyporesponsiveness.

## Methods

### Subjects and measurements

**Figure 1:**
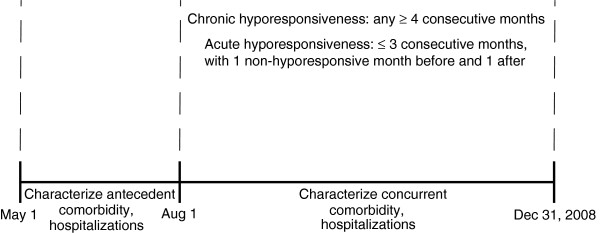
**Study time periods. **The months August through December were used to characterize hyporesponsiveness and comorbidity concurrent with the hyporesponsiveness period. The months May through July were used to characterize comorbidity prior to the hyporesponsive period (antecedent period).

We studied point prevalent in-center and home hemodialysis patients on May 1, 2008, with Medicare as primary payer, who survived through December 31, 2008. Included patients received recombinant human erythropoietin (EPO) in each month, August through December. Figure 1 displays the time periods used in the study. The months August through December were used to characterize hyporesponsiveness and comorbidity concurrent with the hyporesponsiveness period. The months May through July were used to characterize comorbidity prior to the hyporesponsive period (antecedent period).

Three different definitions of hyporesponsiveness were applied to each of the 5 months:

1. Above the ninetieth percentile of total monthly EPO dose.

2. Above the ninetieth percentile of total monthly EPO dose divided by patient weight in kg.

3. Above the ninetieth percentile of total monthly EPO dose divided by patient hemoglobin level (also known as the EPO resistance index).

Definition 2 acknowledges that EPO dosing may depend on patient weight, and definition 3 considers a measure that has been used in other studies to assess issues related to hyporesponsiveness. Table 1 displays the distributions for these definitions. As an example, using total monthly EPO dose, a patient whose total dose was greater than 176,400 units (the ninetieth percentile) was considered hyporesponsive for that month. Each patient in the hyporesponsive category for at least 1 of the 5 months was considered hyporesponsive. Next, considering all 5 months for each patient, hyporesponsive patients were further classified as chronic (hyporesponsive in ≥ 4 consecutive months), acute (hyporesponsive in ≤ 3 consecutive months and not hyporesponsive for at least 1 month before and 1 month after the hyporesponsive months), or other (neither the chronic nor the acute definition applied).

**Table 1:** Distributions of hyporesponsiveness definitions^*^

		**Percentile**					
**Hyporesponsiveness definition**	**Minimum**	**10th**	**20th**	**50th**	**80th**	**90th**	**Maximum**
Monthly EPO dose	500.0	13,800.0	22,100.0	52,000.0	116,000.0	176,400.0	728,000.0
Monthly EPO dose/patient weight in kg	3.4	180.1	291.4	677.1	1534.8	2346.7	476,666.7
Resistance index^†^	34.0	1140.7	1869.2	4442.3	10,361.9	16,252.3	111,815.5

EPO, erythropoietin.

^*^Monthly erythropoietin dose, monthly erythropoietin dose divided by patient weight in kilograms, and monthly erythropoietin dose divided by patient hemoglobin level.

^†^Monthly EPO dose divided by patient hemoglobin level.

Comorbid conditions assessed during the antecedent period were atherosclerotic heart disease, congestive heart failure (CHF), cerebrovascular accident/transient ischemic attack, peripheral vascular disease (PVD), other cardiac disease, chronic obstructive pulmonary disease, gastrointestinal bleeding, liver disease, dysrhythmia, cancer, and diabetes. These conditions were again assessed during the concurrent period. We also characterized age, sex, race, cause of renal failure, dialysis duration, average body mass index during the concurrent period, vascular access complications, intravenous (IV) antibiotic use, number of hospital admissions, number of hospitalization days, infectious hospitalizations, profit status of dialysis provider, and region of the country. Both antecedent and concurrent comorbid conditions were defined from Medicare Part A and Part B claims, using a method previously validated for diabetes [1]. This method uses International Classification of Diseases, Ninth Revision, Clinical Modification diagnosis codes, and requires at least one inpatient, home health, or skilled nursing facility claim with a qualifying diagnosis, or at least two physician/supplier or hospital outpatient claims with a qualifying diagnosis, to define the condition.

### Analysis

Descriptive statistics for categorical variables (count [*n*], percent) were used to examine patient demographic characteristics and comorbid conditions according to hyporesponsiveness definition (total EPO dose, EPO dose/weight in kg, EPO dose/hemoglobin level) and category (chronic, acute, other). Logistic regression was used to assess associations of patient factors with any hyporesponsiveness (chronic, acute, and other). SAS, version 9.1 (SAS Institute Inc., Cary, North Carolina) was used for data analysis.

### Ethics statement

This study was performed with the approval of the Hennepin County Medical Center (Minneapolis, Minnesota) institutional review board.

## Results

The study included 138,688 patients. Patient characteristics were similar to the overall Medicare prevalent hemodialysis population: 52% male, 42% African American, 48% aged ≥ 65 years, 45% with diabetes as cause of renal failure, and 42% with dialysis duration < 3 years.

Table 2 shows the hyporesponsiveness categories obtained using total EPO dose to define monthly hyporesponsiveness, by patient characteristics. The categories are chronic (hyporesponsive for ≥ 4 consecutive months), acute (hyporesponsive for ≤ 3 consecutive months with at least 1 non-hyporesponsive month before and after), and other (some patterns of monthly hyporesponsiveness fit neither the chronic nor the acute categories). Of patients with any hyporesponsiveness, 23%, 26%, and 51% were classified as chronic, acute, or other, respectively. To determine how to classify the “other” group, the squared error for each row of Table 2 was calculated, other compared with chronic, and other compared with acute. The square root of the sum of these values (a measure of distance) confirms that the “other” category is most similar to the acute category (distance = 43.6 for other/chronic, distance = 12.0 for other/acute); therefore, other and acute were grouped into a “non-chronic” category for further analyses. Distance was also assessed using the other 2 definitions of hyporesponsiveness, with similar findings (not shown).

**Table 2:** Hyporesponsiveness categories for total erythropoietin dose

		**Hyporesponsiveness**				**Squared difference**	
	**All**	**None**	**Chronic**	**Acute**	**Other**	**Other - chronic**	**Other - acute**
*n*	138,688	111,422	6248	7219	13,799		
**Sex**							
Men	51.8	51.6	54.7	51.0	52.5	4.9	2.3
Women	48.2	48.4	45.3	49.0	47.5		
**Race**							
White	51.4	52.6	46.2	47.4	46.0	0.1	1.8
African American	42.2	40.5	49.8	47.5	49.5	0.1	4.1
Other	6.4	6.9	3.9	5.1	4.4		
**Age, years**							
< 40	8.1	7.5	12.9	9.8	10.3	7.0	0.2
40-64	44.3	42.7	54.9	48.3	50.3	20.6	4.1
65-74	24.9	25.5	19.8	24.5	22.8	9.3	2.9
≥ 75	22.7	24.4	12.5	17.4	16.6		
**Cause of renal failure**							
Diabetes	44.9	45.3	38.7	45.7	44.1	29.0	2.8
Hypertension	29.3	29.6	27.8	27.8	28.8	1.0	1.1
Glomerulonephritis	10.2	10.0	12.2	10.3	10.7	2.2	0.1
Cystic kidney disease	2.2	2.2	2.1	2.0	1.8	0.1	0.1
Other	13.5	13.0	19.3	14.2	14.7		
**Dialysis duration, years**							
< 1	9.6	9.8	7.7	9.4	8.6	0.9	0.5
1 to < 3	32.6	33.5	26.3	31.3	28.9	6.7	5.8
3 to < 5	23.6	23.6	23.2	23.0	23.8	0.3	0.7
≥ 5	34.3	33.1	42.8	36.4	38.7		
**Average BMI, kg/m**^**2**^							
< 18.5	3.7	3.6	3.3	4.3	3.8	0.2	0.3
≥ 18.5 and < 25	34.9	35.2	30.4	36.5	33.5	9.4	8.8
≥ 25 and < 30	28.5	28.9	25.6	27.6	27.3	2.8	0.1
≥ 30	33.0	32.3	40.7	31.6	35.4		
**Antecedent comorbid conditions**							
ASHD	22.0	20.4	26.0	29.8	28.5	6.3	1.6
CHF	19.6	17.2	29.9	29.7	28.8	1.3	0.8
CVA/TIA	7.4	6.9	7.5	10.9	9.6	4.0	1.9
PVD	16.5	14.8	23.1	24.2	23.3	0.1	0.9
Other cardiac disease	12.4	10.6	20.3	20.0	19.2	1.1	0.6
COPD	8.4	7.3	13.2	12.7	12.9	0.1	0.0
GI bleeding	3.3	2.5	7.2	6.1	6.1	1.2	0.0
Liver disease	5.5	5.2	7.4	6.3	6.8	0.3	0.3
Dysrhythmia	13.7	12.4	19.1	19.2	18.8	0.0	0.1
Cancer	4.1	3.6	7.2	5.3	5.8	2.1	0.2
Diabetes	49.7	48.9	49.2	55.0	53.0	14.1	4.1
**Concurrent comorbid conditions**							
ASHD	30.1	28.4	32.9	41.2	37.0	16.8	17.3
CHF	27.6	24.6	38.9	42.9	39.1	0.0	14.7
CVA/TIA	10.9	10.2	10.5	16.9	13.5	9.3	11.3
PVD	24.8	22.8	30.3	35.8	32.7	5.6	10.0
Other cardiac disease	19.7	17.2	28.3	32.4	28.8	0.3	12.5
COPD	12.5	11.1	18.2	19.2	17.9	0.1	1.6
GI bleeding	5.3	4.1	10.6	11.2	9.6	1.0	2.7
Liver disease	6.4	6.0	8.9	7.8	8.0	0.9	0.0
Dysrhythmia	19.8	18.1	25.1	28.4	26.4	1.8	3.9
Cancer	5.6	5.0	9.9	7.2	7.6	5.5	0.1
Diabetes	57.3	56.7	55.6	62.1	60.2	20.8	3.7
**Number of vascular access complication**							
0	61.9	61.9	64.0	55.4	53.7	106.1	2.7
1-3	19.9	19.9	19.1	21.7	23.4	17.8	2.7
≥ 4	18.2	18.2	16.9	22.9	22.9		
**Average iron dose per month, mg**							
Non-users	15.1	16.2	13.0	9.5	10.4	7.1	0.7
< 233	21.1	23.0	11.4	14.6	13.9	6.6	0.5
≥ 233 and < 360	21.1	21.5	16.6	20.2	20.1	12.0	0.0
≥ 360 and < 460	20.9	20.5	20.9	23.9	22.5	2.5	2.0
≥ 460	21.8	18.8	38.1	31.8	33.2	24.5	1.9
**IV antibiotic use**							
No	76.9	76.9	79.6	65.6	66.6	168.0	1.0
Yes	23.1	23.1	20.5	34.4	33.4		
**Number of hospital admissions**							
0	63.3	63.3	68.3	45.7	44.8	549.0	0.8
1	20.8	20.8	19.4	26.7	26.0	43.2	0.5
≥ 2	15.9	15.9	12.3	27.6	29.2		
**Number of hospitalization days**							
0	63.3	63.3	68.3	45.7	44.8	549.0	0.8
1-7	18.4	18.4	17.3	24.4	22.4	25.6	4.2
≥ 8	18.4	18.4	14.4	29.8	32.8		
**Infectious hospitalizations**							
No	88.2	88.2	90.6	80.3	79.2	129.8	1.2
Yes	11.8	11.8	9.4	19.7	20.8		
**Dialysis provider**							
Non-profit	14.0	14.0	14.7	12.4	11.0	13.0	1.7
For profit	85.0	85.0	84.4	86.5	87.6	9.8	1.1
Unknown	1.0	1.0	0.9	1.1	1.4		
**Region**							
Northeast	15.7	15.7	15.7	15.0	15.1	0.4	0.0
Midwest	19.1	19.1	19.4	19.0	17.8	2.6	1.5
South	45.2	45.2	43.7	50.8	51.9	67.9	1.2
West	17.2	17.2	18.3	12.6	12.6	33.1	0.0
Unknown	2.8	2.8	2.9	2.7	2.7		
Distance	--	--	--	--	--	43.6	12.0

*ASHD*, atherosclerotic heart disease; *BMI*, body mass index; *CHF*, congestive heart failure; *COPD*, chronic obstructive pulmonary disease; *CVA/TIA*, cerebrovascular accident/transient ischemic attack; *GI*, gastrointestinal; *IV,* intravenous; *PVD*, peripheral vascular disease.

Table 3 shows the three definitions of hyporesponsiveness, and within each the categories of non-hyporesponsive, chronic, and non-chronic. A similar number of patients were classified as non-hyporesponsive, chronic, and non-chronic by each of the three definitions, and a comparison of patient characteristics showed no major differences. Men were more likely than women to be chronically hyporesponsive; African Americans were more likely than whites or members of other races to be hyporesponsive generally (chronic and non-chronic). Younger patients were more likely to be hyporesponsive generally, and patients whose cause of renal failure was other than diabetes or hypertension were more likely to be chronically hyporesponsive. Comparing comorbid conditions, all were somewhat more likely when ascertained concurrently with hyporesponsiveness than when ascertained in the antecedent period. Comorbidity burdens were lowest for non-hyporesponsive patients. Comparing chronic with non-chronic hyporesponsiveness with respect to comorbid conditions, levels of comorbidity were generally slightly higher for patients classified with non-chronic hyporesponsiveness, particularly when comorbidity was assessed concurrently with the hyporesponsive period. Patients receiving IV antibiotics were more likely to be classified with non-chronic hyporesponsiveness, as were patients with three or more hospitalizations.

**Table 3:** The three definitions of hyporesponsiveness

	**Monthly EPO dose**				**Monthly EPO dose/patient weight in kg**				Resistance index^*^			
**Hyporesponsiveness type**	**None**	**Chronic**	**Non-chronic**	**Any**	**None**	**Chronic**	**Non-chronic**	**Any**	**None**	**Chronic**	**Non-chronic**	**Any**
*n*	111,422	6248	21,018	27,266	110,767	5905	22,016	27,921	110,022	5761	22,905	28,666
**Sex**												
Men	51.6	54.7	52.0	52.6	52.9	48.3	47.3	47.5	51.6	55.0	51.7	52.4
Women	48.4	45.3	48.0	47.4	47.1	51.7	52.7	52.5	48.4	45.0	48.3	47.6
**Race**												
White	52.6	46.2	46.5	46.4	52.5	45.8	47.2	46.9	52.6	46.4	46.7	46.6
African American	40.5	49.8	48.8	49.1	40.9	48.4	46.7	47.0	40.5	49.6	48.4	48.6
Other	6.9	3.9	4.7	4.5	6.5	5.9	6.2	6.1	6.9	4.0	4.9	4.7
**Age, years**												
< 40	7.5	12.9	10.1	10.7	7.5	13.7	9.8	10.6	7.4	13.3	10.1	10.7
40 to 64	42.7	54.9	49.6	50.8	43.7	49.2	45.8	46.5	42.7	54.8	49.2	50.3
65 to 74	25.5	19.8	23.4	22.6	25.3	20.5	23.8	23.1	25.4	19.4	23.7	22.8
≥ 75	24.4	12.5	16.9	15.9	23.5	16.6	20.5	19.7	24.5	12.5	17.0	16.1
**Cause of renal failure**												
Diabetes	45.3	38.7	44.6	43.3	46.3	32.4	41.3	39.4	45.3	38.3	44.5	43.3
Hypertension	29.6	27.8	28.4	28.3	29.2	29.6	29.9	29.8	29.6	27.7	28.5	28.3
Glomerulonephritis	10.0	12.2	10.6	10.9	9.9	13.2	10.8	11.3	10.2	10.0	12.1	10.5
Cystic kidney disease	2.2	2.1	1.9	1.9	2.2	2.2	2.1	2.1	2.2	2.2	2.1	2.1
Other	13.0	19.3	14.5	15.6	12.5	22.6	16.0	17.4	13.5	12.9	19.8	14.4
**Dialysis duration, years**												
< 1	9.8	7.7	8.9	8.6	9.8	7.5	8.8	8.5	9.8	7.9	8.9	8.7
1 to < 3	33.5	26.3	29.7	29.0	33.9	24.2	28.6	27.7	33.5	26.4	30.0	29.3
3 to < 5	23.6	23.2	23.5	23.4	23.8	21.2	23.1	22.7	23.6	22.9	23.5	23.4
≥ 5	33.1	42.8	37.9	39.0	32.5	47.0	39.5	41.1	33.1	42.9	37.5	38.6
**Average BMI, kg/m**^**2**^												
< 18.5	3.6	3.3	4.0	3.8	2.8	8.3	6.9	7.2	3.6	3.5	4.1	4.0
≥ 18.5 and < 25	35.2	30.4	34.5	33.6	32.2	47.2	44.7	45.2	35.1	31.1	34.8	34.1
≥ 25 and < 30	28.9	25.6	27.4	27.0	29.2	25.1	26.2	26.0	28.9	25.9	27.2	26.9
≥ 30	32.3	40.7	34.1	35.6	35.8	19.4	22.1	21.5	32.4	39.5	33.9	35.0
**Antecedent comorbid conditions**												
ASHD	20.4	26.0	29.0	28.3	20.3	26.8	29.1	28.6	20.3	25.9	28.7	28.1
CHF	17.2	29.9	29.1	29.3	17.0	30.7	29.6	29.8	17.1	29.9	28.8	29.0
CVA/TIA	6.9	7.5	10.0	9.5	6.7	8.7	10.5	10.1	6.9	7.6	9.9	9.4
PVD	14.8	23.1	23.6	23.5	14.9	22.7	23.3	23.2	14.8	23.4	23.3	23.3
Other cardiac disease	10.6	20.3	19.5	19.7	10.4	21.8	20.0	20.4	10.6	20.4	19.0	19.3
COPD	7.3	13.2	12.8	12.9	7.2	13.6	12.9	13.0	7.3	13.4	12.6	12.8
GI bleeding	2.5	7.2	6.1	6.4	2.4	8.0	6.4	6.7	2.5	7.6	6.1	6.4
Liver disease	5.2	7.4	6.6	6.8	5.2	7.7	6.5	6.8	5.2	7.6	6.6	6.8
Dysrhythmia	12.4	19.1	19.0	19.0	12.2	20.0	19.1	19.3	12.4	19.3	18.6	18.7
Cancer	3.6	7.2	5.6	6.0	3.5	8.0	5.6	6.1	3.6	7.6	5.5	5.9
Diabetes	48.9	49.2	53.6	52.6	49.8	43.6	50.4	49.0	48.9	49.3	53.6	52.7
**Concurrent comorbid conditions**												
ASHD	28.4	32.9	38.4	37.2	28.3	33.6	38.4	37.4	28.3	33.2	38.4	37.3
CHF	24.6	38.9	40.4	40.1	24.3	40.2	40.9	40.7	24.4	39.9	40.0	40.0
CVA/TIA	10.2	10.5	14.6	13.7	9.9	11.9	15.5	14.7	10.1	10.7	14.6	13.8
PVD	22.8	30.3	33.7	32.9	22.8	30.5	33.6	32.9	22.6	31.1	33.7	33.2
Other cardiac disease	17.2	28.3	30.0	29.6	16.9	30.9	30.6	30.7	17.0	29.2	29.9	29.8
COPD	11.1	18.2	18.4	18.3	11.0	18.1	18.5	18.4	11.0	19.1	18.2	18.4
GI bleeding	4.1	10.6	10.1	10.2	4.0	11.9	10.2	10.6	4.0	11.4	10.2	10.4
Liver disease	6.0	8.9	7.9	8.1	5.9	9.4	7.9	8.2	6.0	9.3	7.7	8.0
Dysrhythmia	18.1	25.1	27.1	26.6	18.0	26.8	27.1	27.0	18.0	25.8	26.8	26.6
Cancer	5.0	9.9	7.4	8.0	4.9	10.6	7.8	8.4	5.0	10.5	7.4	8.0
Diabetes	56.7	55.6	60.8	59.6	57.7	49.3	57.5	55.8	56.6	55.5	60.8	59.7
**Number of vascular access complication**												
0	61.9	64.0	53.0	55.5	63.7	56.1	54.5	54.8	64.2	55.1	53.0	53.4
1-3	19.9	19.1	23.3	22.4	19.2	21.9	22.7	22.5	19.1	21.8	23.2	22.9
≥ 4	18.2	16.9	23.7	22.1	17.0	22.0	22.9	22.7	16.7	23.1	23.8	23.7
**Average iron dose per month, mg**												
Non-users	16.2	13.0	10.1	10.7	16.0	14.2	11.1	11.8	16.2	13.5	10.4	11.0
< 233	23.0	11.4	14.2	13.5	22.9	12.4	14.9	14.3	23.1	11.4	14.4	13.8
≥ 233 and < 360	21.5	16.6	20.1	19.3	21.5	17.1	20.2	19.5	21.5	16.4	20.3	19.5
≥ 360 and < 460	20.5	20.9	22.9	22.5	20.6	20.7	22.3	21.9	20.6	20.4	22.5	22.1
≥ 460	18.8	38.1	32.7	34.0	19.1	35.5	31.6	32.4	18.7	38.3	32.4	33.6
**IV antibiotic use**												
No	76.9	79.6	66.5	69.5	79.4	66.3	67.5	67.2	79.7	64.9	66.5	66.2
Yes	23.1	20.5	33.5	30.5	20.6	33.7	32.5	32.8	20.3	35.1	33.5	33.8
**Number of hospital admissions**												
0	63.3	68.3	42.1	48.1	68.6	43.6	42.0	42.3	68.7	43.5	42.2	42.5
1	20.8	19.4	26.4	24.8	19.4	26.2	26.7	26.6	19.3	26.7	26.7	26.7
≥ 2	15.9	12.3	31.5	27.1	12.1	30.2	31.4	31.1	12.0	29.8	31.2	30.9
**Number of hospitalization days**												
0	63.3	68.3	42.1	48.1	68.6	43.6	42.0	42.3	68.7	43.5	42.2	42.5
1-7	18.4	17.3	22.1	21.0	17.3	23.8	22.2	22.5	17.2	24.1	22.4	22.7
≥ 8	18.4	14.4	35.8	30.9	14.1	32.6	35.8	35.1	14.1	32.4	35.4	34.8
**Infectious hospitalizations**												
No	88.2	90.6	78.0	80.9	90.7	79.1	78.2	78.4	90.8	78.9	78.3	78.4
Yes	11.8	9.4	22.0	19.1	9.3	20.9	21.8	21.6	9.2	21.1	21.7	21.6
**Dialysis provider**												
Non-profit	14.0	14.7	11.2	12.0	14.6	13.0	11.6	11.9	14.6	12.5	11.7	11.9
For profit	85.0	84.4	87.5	86.8	84.5	85.8	87.1	86.8	84.5	86.4	87.0	86.9
Unknown	1.0	0.9	1.3	1.2	0.9	1.2	1.3	1.3	0.9	1.2	1.3	1.3
**Region**												
Northeast	15.7	15.7	15.4	15.5	15.6	15.6	15.9	15.8	15.8	15.3	15.3	15.3
Midwest	19.1	19.4	17.5	17.9	19.5	17.5	17.2	17.3	19.4	19.0	17.8	18.0
South	45.2	43.7	51.7	49.8	44.0	49.4	50.3	50.1	43.7	50.2	51.4	51.2
West	17.2	18.3	12.9	14.2	18.0	14.8	14.1	14.2	18.3	12.9	13.1	13.1
Unknown	2.8	2.9	2.5	2.6	2.9	2.9	2.4	2.5	2.9	2.6	2.5	2.5

*ASHD*, atherosclerotic heart disease; *BMI*, body mass index; *CHF*, congestive heart failure; *COPD*, chronic obstructive pulmonary disease; *CVA/TIA*, cerebrovascular accident/transient ischemic attack; *EPO*, erythropoietin; *GI*, gastrointestinal; *IV*, intravenous; *PVD*, peripheral vascular disease.

^*^Monthly EPO dose divided by patient hemoglobin level.

Table 4 shows adjusted odds ratios for any hyporesponsiveness compared with none. Two of the three methods showed similar results: total EPO dose and EPO dose divided by hemoglobin level (resistance index). Women, African Americans, and patients aged < 40 years, with cause of renal failure other than diabetes or hypertension, or longer dialysis duration, were more likely to be hyporesponsive. The antecedent comorbid conditions most predictive of any subsequent hyporesponsiveness were CHF, PVD, other cardiac disease, gastrointestinal bleeding, and cancer. Of concurrent comorbid conditions, associations with any hyporesponsiveness were strongest for gastrointestinal bleeding and cancer. Results of EPO dose per kg of body weight were somewhat different with respect to odds ratios for weight and sex; this is not surprising, since in this model, weight would be expected to be a strong predictor of EPO dose divided by weight. The term for sex is also affected since weight is correlated with sex.

**Table 4:** Adjusted odds ratios for hyporesponsiveness

	**Monthly EPO dose**		**Monthly EPO dose/patient weight in kg**		Resistance index^*^	
	**Odds ratio (95% CI)**	***P***	**Odds ratio (95% CI)**		**Odds ratio (95% CI)**	**P**
**Sex**						
Men	1.00		1.00		1.00	
Women	1.08 (1.04-1.11)	< 0.0001	1.49 (1.44-1.54)	< 0.0001	1.08 (1.05-1.11)	< 0.0001
**Race**						
White	1.00		1.00		1.00	
African American	1.11 (1.07-1.15)	< 0.0001	1.07 (1.03-1.11)	< 0.0001	1.10 (1.06-1.14)	< 0.0001
Other	1.01 (0.94-1.08)	0.89	1.22 (1.15-1.30)	< 0.0001	1.04 (0.97-1.12)	0.23
**Age- years**						
< 40	1.00		1.00		1.00	
40 to 64	0.87 (0.82-0.92)	< 0.0001	0.85 (0.81-0.90)	< 0.0001	0.86 (0.81-0.91)	< 0.0001
65 to 74	0.68 (0.64-0.73)	< 0.0001	0.73 (0.68-0.77)	< 0.0001	0.69 (0.65-0.73)	< 0.0001
≥ 75	0.54 (0.51-0.58)	< 0.0001	0.61 (0.57-0.65)	< 0.0001	0.54 (0.51-0.57)	< 0.0001
**Cause of renal failure**						
Diabetes	1.00		1.00		1.00	s
Hypertension	1.11 (1.06-1.16)	< 0.0001	1.12 (1.07-1.17)	< 0.0001	1.12 (1.07-1.17)	< 0.0001
Glomerulonephritis	1.20 (1.13-1.28)	< 0.0001	1.18 (1.11-1.25)	< 0.0001	1.22 (1.15-1.30)	< 0.0001
Cystic kidney disease	1.05 (0.94-1.18)	0.3771	1.03 (0.93-1.15)	0.5768	1.07 (0.96-1.20)	0.2223
Other	1.40 (1.32-1.47)	< 0.0001	1.44 (1.36-1.52)	< 0.0001	1.40 (1.33-1.48)	< 0.0001
**Dialysis duration- years**						
< 1	1.00		1.00		1.00	
1 to < 3	1.13 (1.06-1.19)	< 0.0001	1.05 (0.99-1.11)	0.10	1.13 (1.07-1.19)	< 0.0001
3 to < 5	1.36 (1.29-1.45)	< 0.0001	1.28 (1.21-1.36)	< 0.0001	1.34 (1.27-1.42)	< 0.0001
≥ 5	1.60 (1.51-1.70)	< 0.0001	1.57 (1.48-1.66)	< 0.0001	1.56 (1.47-1.65)	< 0.0001
**Average BMI- kg/m**^**2**^						
< 18.5	1.00		1.00		1.00	
≥ 18.5 and < 25	0.97 (0.90-1.05)	0.48	0.56 (0.52-0.60)	< 0.0001	0.94 (0.87-1.02)	0.12
≥ 25 and < 30	0.99 (0.91-1.07)	0.73	0.35 (0.32-0.37)	< 0.0001	0.94 (0.86-1.02)	0.12
≥ 30	1.05 (0.97-1.14)	0.23	0.20 (0.18-0.21)	< 0.0001	0.99 (0.91-1.07)	0.79
**Antecedent comorbid conditions**						
ASHD	1.08 (1.04-1.13)	0.0002	1.11 (1.06-1.15)	< 0.0001	1.08 (1.04-1.13)	0.0002
CHF	1.23 (1.18-1.28)	< 0.0001	1.24 (1.19-1.29)	< 0.0001	1.24 (1.19-1.29)	< 0.0001
CVA/TIA	1.05 (0.99-1.11)	0.09	1.07 (1.01-1.13)	0.02	1.05 (0.99-1.11)	0.12
PVD	1.25 (1.20-1.30)	< 0.0001	1.21 (1.17-1.26)	< 0.0001	1.24 (1.19-1.29)	< 0.0001
Other cardiac disease	1.25 (1.20-1.31)	< 0.0001	1.27 (1.21-1.32)	< 0.0001	1.21 (1.16-1.26)	< 0.0001
COPD	1.18 (1.12-1.25)	< 0.0001	1.16 (1.10-1.23)	< 0.0001	1.16 (1.10-1.23)	< 0.0001
GI bleeding	1.51 (1.40-1.62)	< 0.0001	1.62 (1.51-1.74)	< 0.0001	1.56 (1.45-1.68)	< 0.0001
Liver disease	1.17 (1.07-1.28)	0.00	1.13 (1.04-1.24)	0.01	1.19 (1.09-1.30)	< 0.0001
Dysrhythmia	1.16 (1.10-1.21)	< 0.0001	1.15 (1.09-1.20)	< 0.0001	1.14 (1.09-1.19)	< 0.0001
Cancer	1.45 (1.33-1.57)	< 0.0001	1.36 (1.26-1.48)	< 0.0001	1.43 (1.32-1.55)	< 0.0001
Diabetes	1.07 (1.02-1.13)	0.00	1.06 (1.01-1.11)	0.02	1.09 (1.04-1.14)	0.00
**Concurrent comorbid conditions**						
ASHD	0.94 (0.90-0.97)	0.00	0.93 (0.90-0.97)	0.00	0.94 (0.90-0.97)	0.00
CHF	1.12 (1.08-1.17)	< 0.0001	1.14 (1.09-1.18)	< 0.0001	1.10 (1.06-1.15)	< 0.0001
CVA/TIA	0.94 (0.90-0.99)	0.02	0.99 (0.94-1.04)	0.59	0.94 (0.89-0.99)	0.01
PVD	1.02 (0.98-1.06)	0.25	1.03 (1.00-1.07)	0.08	1.03 (1.00-1.07)	0.09
Other cardiac disease	1.14 (1.10-1.18)	< 0.0001	1.16 (1.12-1.20)	< 0.0001	1.15 (1.10-1.19)	< 0.0001
COPD	1.04 (0.99-1.09)	0.15	1.01 (0.96-1.06)	0.63	1.04 (1.00-1.09)	0.08
GI bleeding	1.45 (1.36-1.53)	< 0.0001	1.44 (1.35-1.52)	< 0.0001	1.50 (1.41-1.59)	< 0.0001
Liver disease	1.05 (0.97-1.14)	0.24	1.09 (1.01-1.19)	0.03	1.01 (0.93-1.09)	0.86
Dysrhythmia	1.03 (0.98-1.07)	0.21	1.00 (0.96-1.04)	0.87	1.02 (0.98-1.07)	0.31
Cancer	1.32 (1.23-1.42)	< 0.0001	1.36 (1.27-1.47)	< 0.0001	1.32 (1.23-1.41)	< 0.0001
Diabetes	1.02 (0.97-1.07)	0.38	0.98 (0.93-1.03)	0.39	1.02 (0.98-1.07)	0.35
**Number of vascular access complication**						
0	1.00		1.00		1.00	
1-3	1.10 (1.06-1.15)	< 0.0001	1.07 (1.03-1.11)	0.0005	1.10 (1.06-1.14)	< 0.0001
≥ 4	1.15 (1.10-1.20)	< 0.0001	1.13 (1.08-1.17)	< 0.0001	1.16 (1.11-1.21)	< 0.0001
**Average iron dose per month- mg**						
Non-users	1.00		1.00		1.00	
< 233	1.00 (0.94-1.06)	0.9360	0.97 (0.92-1.03)	0.3598	0.97 (0.91-1.02)	0.2483
≥ 233 and < 360	1.31 (1.23-1.38)	< 0.0001	1.25 (1.18-1.32)	< 0.0001	1.28 (1.21-1.35)	< 0.0001
≥ 360 and < 460	1.55 (1.47-1.64)	< 0.0001	1.45 (1.38-1.53)	< 0.0001	1.48 (1.40-1.56)	< 0.0001
≥ 460	2.36 (2.24-2.50)	< 0.0001	2.19 (2.07-2.31)	< 0.0001	2.27 (2.15-2.39)	< 0.0001
**IV antibiotic use**						
No	1.00		1.00		1.00	
Yes	1.26 (1.22-1.31)	< 0.0001	1.26 (1.22-1.31)	< 0.0001	1.26 (1.22-1.31)	< 0.0001
**Number of hospital admissions**						
0	1.00		1.00		1.00	
1	1.49 (1.43-1.56)	< 0.0001	1.51 (1.45-1.58)	< 0.0001	1.54 (1.48-1.60)	< 0.0001
≥ 2	1.88 (1.77-1.98)	< 0.0001	1.91 (1.81-2.02)	< 0.0001	1.95 (1.84-2.06)	< 0.0001
**Infectious hospitalizations**						
No	1.00		1.00		1.00	
Yes	1.10 (1.05-1.15)	< 0.0001	1.13 (1.08-1.18)	< 0.0001	1.10 (1.05-1.15)	< 0.0001
**Dialysis provider**						
Non-profit	1.00		1.00		1.00	
For profit	1.19 (1.10-1.28)	< 0.0001	1.19 (1.10-1.28)	< 0.0001	1.18 (1.09-1.27)	< 0.0001
Unknown	1.56 (1.33-1.83)	< 0.0001	1.57 (1.34-1.85)	< 0.0001	1.49 (1.27-1.75)	< 0.0001
**Region**						
Northeast	1.00		1.00		1.00	
Midwest	0.89 (0.85-0.94)	< 0.0001	0.88 (0.84-0.93)	< 0.0001	0.92 (0.87-0.97)	0.0013
South	1.16 (1.11-1.21)	< 0.0001	1.14 (1.09-1.19)	< 0.0001	1.17 (1.12-1.23)	< 0.0001
West	0.80 (0.75-0.84)	< 0.0001	0.83 (0.79-0.88)	< 0.0001	0.81 (0.77-0.86)	< 0.0001
Unknown	0.91 (0.82-1.00)	0.0576	0.90 (0.82-1.00)	0.0401	0.88 (0.80-0.97)	0.0116

*ASHD*, atherosclerotic heart disease; *BMI*, body mass index; *CHF*, congestive heart failure; *CI*, confidence interval; *COPD*, chronic obstructive pulmonary disease; *CVA/TIA*, cerebrovascular accident/transient ischemic attack; *EPO*, erythropoietin; *GI*, gastrointestinal; *IV*, intravenous; *PVD*, peripheral vascular disease.

^*^Monthly EPO dose divided by patient hemoglobin level.

## Discussion

We found very similar associations between patient characteristics and three methods of characterizing EPO hyporesponsiveness (total EPO dose, EPO dose per kg of body weight, or EPO dose per hemoglobin level). Given this finding, using the simplest of the three methods we investigated, total EPO dose, to characterize hyporesponsiveness seems justifiable. Our findings relating hyporesponsiveness to patient characteristics are largely in agreement with other studies in other populations that used different methods to characterize hyporesponsiveness. With respect to the proportion of patients classified as hyporesponsive, our definitions produced approximately 20%, 4.5%, and 15% of patients with any, chronic, and non-chronic hyporesponsiveness, respectively. Other studies have not separated chronic from non-chronic hyporesponsiveness. Attalah et al [2] found that 17.6% of patients were hyporesponsive, defined as requiring > 450 U/kg/week IV EPO, with average 3-month hemoglobin < 11 g/dL, ferritin ≥ 500 ng/mL, and TSAT ≤ 50%. Johnson et al [3] describe approximately 5% to 10% of patients as hyporesponsive, defined as requiring > 450 U/kg/week IV EPO.

Potential causes of hyporesponsiveness have been previously investigated and described, with varying levels of supporting evidence [3-9]. Causes include absolute or functional iron deficiency, inflammation, infection, lack of dialysis adequacy, hyperparathyroidism, hemolysis, nutritional factor deficiencies, aluminum overload, pure red cell aplasia, malignancy, bone marrow disorders, myelosuppressive agent use, and use of angiotensin converting enzyme inhibitors and angiotensin receptor blockers.

Using data from a large dialysis organization, Kalantar-Zadeh et al [10] examined predictors of hyporesponsiveness. Although they used a measure of hyporesponsiveness different from ours (fitting hemoglobin trajectories to individuals with mixed models), and their primary focus was on iron markers and osteodystrophy, their findings were similar with respect to age, sex, race, and major comorbid conditions, including cancer, CHF, and diabetes. One notable difference was with respect to dialysis duration; we found increasing hyporesponsiveness with longer dialysis duration, compared with their findings of decreased hyporesponsiveness. This may be due to the particular categorization of dialysis duration analyzed. The first 6 months after dialysis initiation, and in particular, the first 3 months, is a period of high risk. The mortality hazard appears to be highest shortly after initiation, and decreases during the first 6 months. Correspondingly, dialysis duration beyond 5 years shows further increasing risk, as patients continue to accumulate comorbidity. Therefore, investigation of hyporesponsiveness associations within the first year, and beyond 5 years, may be important for further study.

In another study by Kalantar-Zadeh et al [11], the authors found associations between inflammatory markers and hyporesponsiveness. We did not have access to data on inflammatory markers; however, our findings of higher comorbidity burden, higher number of vascular access complications, and IV antibiotic use are in general agreement with the earlier findings, since inflammatory burden generally increases with these factors.

Limitations of the current study include lack of laboratory data; somewhat arbitrary definitions of hyporesponsiveness based on the ninetieth percentile of total monthly EPO dose, dose/kg, or dose/hemoglobin level; possible misclassification of comorbid conditions due to use of claims to identify them; lack of data on catheter use, which is known to be associated with increased risk of infection and inflammation; and residual confounding, which may explain some of the adjusted logistic regression findings. With respect to laboratory data, some definitions of hyporesponsiveness in the literature have included information on ferritin and TSAT; these data were not available in our study. Despite these unavailable data, our findings are generally similar to findings of other studies. Another limitation is that knowledge of the distribution of hemoglobin levels in a similar, large population is needed to define a ninetieth percentile. Simply examining the distribution in a small population (for example, at a particular dialysis facility) to find the appropriate cut point, which may not include any hyporesponsive individuals, would not be appropriate.

A potential limitation involves the 2008 timeframe of the data. This period is after release of CHOIR [12] and CREATE [13] study results, which produced a change in ESA dosing and hemoglobin levels, and before implementation of the Medicare reimbursement bundle, which may cause further declines in ESA doses and hemoglobin levels. However, use of the ninetieth percentile cut would still identify patients receiving the highest 10% of doses, given the overall population dosing levels at a point in time.

## Conclusions

Associations were similar between patient characteristics/comorbidity and three methods of characterizing EPO hyporesponsiveness. Results using the simplest of the three methods (ninetieth percentile of EPO dose) were also largely similar to other studies, suggesting the utility of the simple method for characterizing hyporesponsiveness. With implementation of the Medicare dialysis reimbursement bundle in January 2011, which includes all injectables, the previous financial incentive to use higher doses of ESAs will be reversed. Anemia management strategies regarding the relative use of ESAs and IV iron therapy may change as a result of this, and how it will affect ESA hyporesponsiveness is unknown. Future studies should continue to investigate this area as anemia management changes in response to the bundle.

## Abbreviations

CHF: Congestive heart failure; PVD: Peripheral vascular disease; EPO: Recombinant human erythropoietin; ESA: Erythropoiesis-stimulating agent; IV: Intravenous.

## Competing interests

This study was supported by a research contract from Takeda Pharmaceuticals International, Inc., Deerfield, Illinois. The contract provides for the authors to have final determination of manuscript content. Yi Peng has no conflicts of interest. Dr. Gilbertson has provided consultation to Amgen, DaVita Clinical Research, and Affymax. Dr. Arneson has an ownership interest in Johnson & Johnson. Stephan Dunning has provided consultation to Amgen. Dr. Collins has provided consultation to Merck, Amgen, Takeda, and NxStage.

## Authors’ contributions

All authors participated in study conception or design, or analysis and interpretation of data, or both; DG drafted the manuscript and YP, TJA, SD and AJC provided intellectual content of critical importance to the work. All authors approved the version to be submitted.

## Pre-publication history

The pre-publication history for this paper can be accessed here:

http://www.biomedcentral.com/1471-2369/14/44/prepub
